# Buffalo Medical Association

**Published:** 1860-01

**Authors:** W. H. Butler


### ART. III.—*Buffalo Medical Association—November Meeting.* Reported by W. H. Butler, M. D., Secretary.

### PUERPERAL CONVULSIONS.—ULCERATION OF THE CORNEA ON APPROACH OF DEATH, &c.

Dr. White reported a case of puerperal convulsions occurring at the Buffalo Lying-in Asylum, as taken from the notes of he interne, Dr. N. S. Lockwood. The patient, E. T., aged 20, unmarried, strong and well-formed, first pregnancy, was taken with labor Oct. 26, at 1½ o'clock, P. M., and was delivered at 12½ the same night. The placenta came away in five minutes after the child; when she was seized with a violent convulsion. Cold was applied to the head, and chloroform administered. After the convulsion, the bandage was applied, and chloroform continued freely. An attempt to give opiates was made, but this failed. In the morning, 1 drop croton oil every hour; spirits of nitre, 1 teaspoonful every four hours; cold to head, counter-irritation to extremities; pulse, 117. Continued the chloroform when convulsions seemed coming on. These ceased at 1, P. M. Whole number of seizures, 31. An injection of castor-oil given at 12 o'clock, produced copious dejection at 1. The following night she had morphine, ¼ gr. every four hours. Tablespoonful brandy, every four hours; the administration of chloroform when necessary. Oct. 28, the patient was ordered seven grains of Dover's powder every four hours, and a teaspoonful spirits of nitre every four hours. The pulse at that time was 122. Five, P. M., half a drachm of carbonate of ammonia dissolved in ℥ iij. water, to take 1 tablespoonful every two hours, was ordered, with 1 tablespoonful of brandy every alternate two hours; beef essence every hour. Pulse 122 and feeble. The albumen in the urine diminishing. The same treatment was continued on the 29th; pulse, 100. On the 30th, quinine, grs. ij., was given every four hours. The same treatment was pursued on the 31st and on Nov. 1st, the pulse being on the 31st, 90, and on the last day 86; the patient now out of danger.

Dr. White thinks puerperal convulsions a disease *sui generis*, not apoplectic, nor epileptic; hence, bleeding is seldom necessary. He considered the case interesting, as showing the beneficial effect of chloroform in lessening the violence of the convulsions, but more especially as showing that plethoric patients recover without bleeding, after many convulsions. It was evident that the woman had no apoplexy, as she recovered so quickly, and, then, there are no convulsions attending apoplexy. Although the patient was stout and plethoric, such a one as all advocates of bleeding would have considered as peculiarly requiring free depletion; and although coma simulating apoplectic coma, lasted many hours, yet did the result demonstrate that no effusion was present. It is not unlikely that copious bleeding would have increased the debility and lessened the chances of her recovery.

The puerperal convulsion is, no doubt, caused by some remote uterine irritation—perhaps uremia, though the latter is not constant. This condition tends to develop convulsions. His theory is, that we should give chloroform and anodynes to relieve this irritable state of the system, to be followed with croton oil as a counter-irritant. This is easily administered, and acts as a powerful revulsive. He thought we should never arrive at a correct theory, or satisfactory treatment of this disease until we change our notions in relation to the character of the seizures. Of four cases he had had in the last summer, three had recovered.

Dr. Burwell remarked that he did not think much could be claimed for the remedy if it did not completely control the seizures. He had never seen a patient with symptoms resembling apoplexy; they appear more like epilepsy—with frothing at the mouth, &c.

Dr. Newman called the attention of the members to a sign of death, or approaching death. This is the ulceration of the cornea, producing the glazed appearance of the eye. He had that day seen a child with convulsions, which he noticed had ulceration of the cornea. He had predicted its death, which had taken place that afternoon. He remarked the same during the prevalence of the cholera here; when the cornea became ulcerated, death was sure to follow. Any exhaustive disease might produce it. This ulceration occurs nearest the center of the cornea. He had often seen the cornea ulcerate and dry up before death, in cholera patients.

Dr. Rochester had been familiar with a muco-purulent deposit that occurs on the eye, though he had not regarded it as ulceration.

Dr. Newman remarked that he had seen the eye-ball nearly shrivel up.

Dr. Cronyn inquired if it did not occur, in some forms of peritonitis, that the anterior chambers of the eye were filled with pus?

Dr. Rochester had been interested and instructed in the able report of Dr. White. He supposed, however, that there was always danger of effusion of serum within the cerebral ventricle, and thinks the effect of drastic cathartics is to prevent or relieve this effusion.

Drs. Rochester, Wyckoff, and Ring, proposed reporting cases at future meetings.

Dr. Newman wanted some facts in relation to puerperal convulsions brought out. There is a theory that effusions into the brain do not produce convulsions; that it requires irritation of the investing membrane of the spinal cord. This, he thinks, militates against the congestive theory.

Dr. Miner thought chloroform might have the same effect as veratrum viride; it lessens the frequency of the pulse, and why not act as a depleting agent?

Dr. Newman had thought, perhaps the chloroform might act on the kidneys to produce such action as to eliminate the urea.

Dr. Gay inquired if Dr. Miner thought chloroform a stimulant or sedative? Dr. Miner replied that externally, it acted as a stimulant; internally, as a sedative.

Dr. Cronyn said it would be interesting to know in what proportion of cases that albumen is in excess in puerperal convulsions, urea is also present. This uremic poison, it is known, tends to develop convulsions.

Dr. Newman then read the following Address:—

### DR. NEWMAN'S ADDRESS.

*Gentlemen of the Association*:—

I little thought when you conferred upon me the honors of the Presidency of this Association, that ere the year expired there would come a time of leave-taking, and that the associations of many a long year would be disruptured. But life is transitory, and so are all its interests. To-night I meet you for the last time. Circumstances unforeseen a year since, lead me to seek a new home.

I come to-night to bid you farewell. Your kindness has delayed the evening of separation, but the hour has finally come when the parting-word must be spoken.

It may be that I degrade my manhood in saying, that the separation gives to me inexpressible pain, and that many an hour of poignant sorrow has been experienced in view of the disrupture of the many pleasant associations connected not only with yourselves, as a society, and the consequent professional intercourse; but the many other attachments which a life from early boyhood has developed and fastened. If it be a degradation to sorrow over the past and all their attachments, I confess to the weakness, and assure you that to part with you has been the source of a severe struggle. I have an attachment perfectly child-like for this city. Coming here at the age of nine years, when its population was but some nine or ten thousand, a residence of twenty-six years has made me a witness of almost every stone that has been planted in the streets, and the placing of every brick which has built up the magnificent edifices of the city. I have gloried in the town. It was the place of my boyhood. All the memories which cluster around my advance from boyhood to manhood are here centered. Some of the memories of the past cost me many a tear. I leave behind me as I go, a mother's grave.

Our Association,—for I do not wish to feel it is not *ours* until I am beyond the precincts of the State,—I have known from its inception. It was organized when I was a student. Accidentally it was my privilege to be present at some of its early meeting. As a student I recognized its importance; need I assure you that as a practitioner I have never lost sight of its value to the profession?

You will bear me witness, that I have been ever willing to labor in its behalf. Whatever may be the value of these labors, they were cheerfully rendered; and if any error has been committed, excuse the want of judgment, in the assurance of the honest zeal in behalf of the interests of the Association, which prompted their exercise. And permit me to assure you, Gentlemen, that the Buffalo Medical Association enjoys to-day a reputation in the land which need bring a blush to no member of the Association. It is considered a working institution, and its members are looked upon as live working men. Our transactions have ever been read with interest. Those, who have contributed to the interest of our meetings have lost nothing in reputation. Without this organization the profession of the city would be really afloat. I have had opportunities of knowing that outside of the city this Association has a reputation as real as it has been well earned.

And now, Gentlemen, your expression last evening was, that I was to leave you with a Valedictory Address. Upon what subject, in parting, can I speak? Shall I speak of our profession? It is a subject beyond the panegyric of my poor pen. To stand at the bed-side as an intelligent physician, and watch day by day the progress of disease, and mark the conflict between your remedies and the disease, and feel that the responsibilities of a life are in your hand, commands every faculty of an earnest soul. But there is an intense and inexpressible interest to the real physician in the observation of the struggle. No labor is too great to secure the victory in the contest. But you know the history of every day's life of the physician.

Shall I speak of the heroism of the profession? The history of epidemics attests it better than I am able. Witness the pestilential emigrant-ship; the lazar-houses of fever; the dens of cholera; the places where men's faces blanch as they enter, but where the physician with a higher and more noble courage than the soldier, faces death, confronts a hidden enemy whose aim is as deadly as the rifle. But alas! the dead physician no one remembers. The victim at the cannon's mouth is the subject of a nation's mourning. The physician dies, and is gathered to his fathers. Who, outside of the profession, know aught of the forty martyrs of Norfolk's yellow-fever epidemic?

But why detail these things at the expense of your time? Ours is a profession of labor—unappreciated too often—abused more frequently than appreciated.

Somber thoughts intrude themselves, at times, upon a review of the past. The quack is too often preferred to the physician. Will it ever be otherwise?

In conclusion, Gentlemen, I thank you for the many expressions of kindness towards me which you have manifested, and for the uniform courtesy I have received at your hands. I regret that an hour of separation has come; but be assured that I shall take with me, and preserve, a most hearty interest in the welfare of this Association, and be as willing to promote its interests when absent, as I have been while with you. Farewell!

Remarks complimentary to Dr. Newman, and expressive of regret at his removal from Buffalo, were then made by Drs. Rochester and White.

Dr. WHITE moved that a committee of three be appointed to accept the resignation of Dr. Newman, and indicate what action should be taken in relation to it.

The Chair appointed as such committee, Drs. White, Miner, and Eastman.

